# Combined cART including Tenofovir Disoproxil, Emtricitabine, and Dolutegravir has potent therapeutic effects in HIV-1 infected humanized mice

**DOI:** 10.1186/s12967-021-03120-w

**Published:** 2021-10-30

**Authors:** Matthew Weichseldorfer, Yvonne Affram, Alonso Heredia, Zahra Rikhtegaran-Tehrani, Mohammad M. Sajadi, Sumiko P. Williams, Yutaka Tagaya, Francesca Benedetti, Habib O. Ramadhani, Frank Denaro, Arshi Munawwar, Joseph Bryant, Davide Zella, Marvin Reitz, Fabio Romerio, Olga S. Latinovic

**Affiliations:** 1 Institute of Human Virology, School of Medicine, University of Maryland, Baltimore, MD 21201 USA; 2 Department of Medicine, School of Medicine, University of Maryland, Baltimore, MD 21201 USA; 3 Department of Biochemistry and Molecular Biology, School of Medicine, University of Maryland, Baltimore, MD 21201 USA; 4 Morgan State University, College of Bio Sciences, Baltimore, MD 21011 USA; 5 Department of Microbiology and Immunology, School of Medicine, University of Maryland, Baltimore, MD 21201 USA; 6 Department of Microbial Pathogenesis and Immunology, University of Texas A and M Health Science Center, Bryan, TX 77843 USA; 7 Department of Molecular and Comparative Pathobiology, Johns Hopkins University School of Medicine, Baltimore, MD 21201 USA

**Keywords:** HIV/AIDS pathogenesis, Hu-mouse models, Antiretroviral therapies

## Abstract

HIV-1 reservoirs persist in the presence of combined antiretroviral therapy (cART). However, cART has transformed HIV-1 infection into a chronic disease marked by control of HIV-1 viral load and mortality reduction. Major challenges remain, including viral resistance upon termination of cART and persistence and identification of tissue distribution of HIV-1 reservoirs. Thus, appropriate animal models that best mimic HIV-1 pathogenesis are important, and the current study complements our previously published validation of the CD34+ hematopoietic humanized mouse model for this purpose. Here we analyze viral suppression using the recently developed combination of antiretrovirals that include Tenofovir Disoproxil (TDF), Emtricitabine (FTC), and Dolutegravir (DTG), a choice based on recent clinical outcomes showing its improved antiretroviral potency, CD4+ T cell preservation, tolerability, and prevention of viral drug resistance compared to that of previous regimens. We used quantitative Airyscan-based super resolution confocal microscopy of selected mouse tissues. Our data allowed us to identify specific solid tissue reservoirs of human T cells expressing the HIV-1 core protein p24. In particular, lymph node, brain, spleen, and liver were visualized as reservoirs for residual infected cells. Marked reduction of viral replication was evident. Considering that detection and visualization of cryptic sites of HIV-1 infection in tissues are clearly crucial steps towards HIV-1 eradication, appropriate animal models with pseudo-human immune systems are needed. In fact, current studies with humans and non-human primates have limited sample availability at multiple stages of infection and cannot easily analyze the effects of differently administered combined antiretroviral treatments on multiple tissues. That is easier to manage when working with humanized mouse models, although we realize the limitations due to low human cell recovery and thus the number of cells available for thorough and comprehensive analyses. Nonetheless, our data further confirm that the CD34+ humanized mouse model is a potentially useful pre-clinical model to study and improve current anti-HIV-1 therapies.

**Supplementary Information:**

The online version contains supplementary material available at 10.1186/s12967-021-03120-w.

## Background

HIV-1 can establish a stably integrated, non-productive latent state of infection of individual cells, mainly in long lived CD4+ T cells that are maintained by homeostatic proliferation [1, 2]. Even with the evident success of cART [3], the latent existing stable reservoirs are prime barriers to HIV-1 cure. Although cART effectively suppresses HIV-1 replication, the persistent latent reservoirs of myeloid [4, 5] and T cells in patients are existing issues towards the goal of HIV-1 eradication [3, 6–8]. HIV-1 can also replicate in brain microglial cells, which persist despite cART [9, 10]. Honeycutt et al. [11] reported that integrated HIV-1 DNA is present in human bone marrow and spleen macrophages even after cART treatment, and that mice with only human myeloid cells allow persistent infection in macrophages during cART in vivo*.*

Mechanisms of latency in CD4+ T cells in vivo are still not fully understood. In addition, better characterization of the tissues that contain cells hosting persistent virus as well as development of novel, more sensitive assays for total DNA analyses are needed for viral eradication studies [12, 13]. Since transformed cell lines used to study HIV-1 latency do not mimic the quiescent cellular environment of primary latently infected cells in vivo, humanized murine models have been proposed for characterization of HIV-1 reservoirs [14–16]. These models provide a platform for testing strategies to eliminate HIV-1 latent reservoirs and prevent viral rebound. Appropriate humanized models require proper humanization procedures of the animals as well as the use of immunodeficient recipient mouse strains that harbor various mutations [17]. An example is the SCID mouse [18, 19], in which a mutation in the catalytic polypeptide subunit (*Prkdc*^*scid*^) of the gene that encodes the DNA-activated protein kinase (DNA-PK) prevents efficient DNA repair required for T and B cell receptor rearrangement. This in turn results in a lack of circulating B and T lymphocytes. One use of such models (with immune features translatable to human) is to provide comprehensive information on different tissues during oral cART therapy, which is obviously not feasible in infected humans.

The small number of latently infected cells (~ 1 in 1 × 10^6^ resting CD4+ T cells) and lack of surface latency markers make detection of HIV-1 reservoirs after antiretroviral treatment very challenging [20]. cART refractory latent reservoirs are the primary reason for the rapid virus rebound that occurs when latent cells become reactivated in humans [21], and their presence precludes complete eradication with the cART options available today. Additionally, cART is further unable to fully suppress HIV-1 replication due to poor drug penetration into certain tissues, such as the central nervous system [22].

Our previous study laid a foundation for the use of HIV-1 infected hu-NSG mice to test the effects of cART, alone or in combination with CCR5 targeting drugs, on productive HIV-1 viral replication [23]. We showed that the hu-NSG model mimics key aspects of HIV-1 pathogenesis in vivo, with HIV-1 reservoirs that are sequestered from the immune system and antiretroviral drugs. The current study looks at a recently developed cART cocktail, consisting of Tenofovir Disoproxil (TDF), Emtricitabine (FTC), and Dolutegravir (DTG) [24], using the same model and methodology as our previous studies to validate its efficacy in a humanized mouse model. In treatment-experienced patients, cART regimens based on once-daily DTG showed greater viral suppression when compared to twice-daily RAL (71% DTG versus 64% RAL) [25, 26]. McAllister et al. showed that DTG with TDF and FTC is a well-tolerated option for once daily postexposure in MSM [27]. In addition, DTG exhibits a higher barrier to resistance than RAL. It has a low interaction potential, so there are no food restrictions [28–30]. Based on the most recent Guidelines for the Use of Antiretroviral Agents in Adults and Adolescents with HIV-1 [31], DTG has become a recommended component in combined and recommended initial regiments for most people with HIV-1.

The hu-NSG model is an effective and valuable surgery-free in vivo system for evaluation of HIV-1 replication, suppression, and dynamic therapy response. In addition, this model ensures that the number of available CD4+ T cells is comparable to that of HIV-1 patients under cART therapy [18]. This study allows more thorough analyses of a set of samples not readily available from human subject studies.

## Materials and methods

### NSG newborn mouse engraftment with human cord blood CD34+ cells

Newborn animals were transplanted with 1 × 10^5^ human CD34+ hematopoietic stem cells obtained from umbilical cord blood as previously described [14, 18, 23, 32] (Lonza, donor 28753, Cat No. #2C-101). Newborns 3–4 days old were irradiated with 100 cGy and injected intrahepatically with CD34+ cells within 3 h after irradiation. CD34+ cells were thawed and assessed for viability before injections. Animals were housed under pathogen-free conditions at the IHV Animal Facility, School of Medicine, University of Maryland (SOM UM) Baltimore, Maryland. All experimental protocols were in accordance with the NIH guide for the care and use of laboratory animals and approved by the SOM UM IACUC. Twenty weeks after CD34+ HSC transplantation, mice were selected based on the expansion of human CD45+ cells with T>B cells (CD3+ cell numbers higher than CD19+ cell numbers), as judged by flow cytometry. Animals with less than a minimum of 20% human CD3+ T cells were not selected for this study [23, 32, 33]. The 13 mice selected were divided into three experimental groups: 8 to be infected intraperitoneally with 10,000 TCID_50%_ of HIV-1 BaL (200 µL total volume per mouse) at around 20 weeks of age, of which 5 mice were to be treated with oral cART for 17 weeks, and 5 mice to remain uninfected as a control group. HIV-1 BaL, an R5 replication competent virus that contains most of the BaL *env* gene in an HIV-1 IIIB backbone, titer 1 × 10^5^/mL [34], was used to infect 8 mice. Viral stocks were provided by the µQuant Core Lab, IHV SOM UM. The NIH regulation [35] mandating inclusion of both mouse genders in experimental groups was respected; each experimental group had sex ratios of 1:1. To monitor graft versus host disease (GVHD) that could interfere with the experimental outcome, the animals were closely observed for hair and weight loss, as they are the main indications of GVHD. No hair changes or weight reduction higher than 10% were observed. Mice were euthanized at the end of week 20 according to the IACUC protocol regulations.

### Treatment with antiretroviral drugs (cART)

Doses for standard cART were chosen based on the published therapeutic efficacy in chronic HIV-1 infection in humans [36, 37] adjusted for mouse body weights. Food pellets (Mod TestDiet) containing cART [a mixture of Tenofovir Disoproxil (TDF), Emtricitabine (FTC) and Dolutegravir (DTG)] were administered to mice daily for 17 weeks. Cylindrical pills were manufactured as single doses of 60 mg TDF, 60 mg FTC, and 48 mg DTG per kg of food. Pellets also contained antibiotic (amoxicillin, 0.12%) and were irradiated before use. Food pellets containing cART mix were orally administered daily to each mouse. Mice were monitored for drug intake and given regular food pellets after finishing the medicated pellets. Mice were weighed twice per week and monitored for fur loss or change of behavior twice daily. Fresh water was changed every 2 days and always available ad libitum.

### Staining procedures for confocal microscopic visualization of human lymphocytes and HIV-1 structural protein, p24

Primary PBMCs isolated from donor whole blood were cultured and activated from frozen stocks for 3 days prior to infection, then split into infected and non-infected groups, with the infected group being cultured with HIV BaL (MOI = 0.00075) for 3 h at 37 °C 5% CO_2_. Afterwards, media was replaced and both groups cultured separately for 1 week. Infected and non-infected primary cells were stained using rabbit α-CD4 (Invitrogen, Cat. MA5-16338) primary antibody at a 1:25 dilution followed by goat α-rabbit AF647 (Abcam, Cat. ab150079) secondary antibody at a 1:250 dilution for 45 min each at 4 °C. Cells were fixed and permeabilized with Foxp3 Fixation and Permeabilization Buffer (Invitrogen, Cat. 00-5523-00) for 45 min at 4 °C, then stained using mouse α-p24 FITC (Beckman Coulter, Cat. 6604665) at 1:25 for 45 min at 4 °C followed by 50 µg/mL DAPI for 1 min. Cells were imaged in 1% PFA diluted in PBS. Controls were stained without rabbit α-CD4 primary and/or with mouse IgG1 Alexa Fluor 488 (Biolegend, Cat. 400134) in place of α-p24 FITC. HIV-1 negative and control cells showed no positive staining (not shown).

For tissue imaging, slides were warmed (45 °C) for at least 10 min to remove paraffin, then submerged in xylene. Containers with slides were placed into dehydration buckets containing 100% ethanol and incubated for a few minutes. The procedure was repeated several times with lower concentrations of ethanol, and the slides were then incubated at 95 °C in Dako Target Retrieval Solution (Dako, Cat No. S2368) for 20 min and cooled per the manufacturer’s instruction. Blocking was overnight at 4 °C with 20% BSA in 1× PBS. Surface marker staining used 1:25 α-CD4 Alexa Fluor 488 (Stemcell Technologies, Cat. 60016AD), 1:25 primary antibody α-CD45 (Leica BioSystems, Cat. NCL-L-LCA), or 1:25 primary antibody α-CD68 (Dako, Cat. M0814) for 1 h at room temperature (RT). Slides stained against CD45 or CD68 were then incubated with 1:250 secondary antibody α-mouse IgG (H + L) DyLight 488 (Vector, Cat. DI-2488) for 1 h at RT. Slides were permeabilized with 0.1% Triton X-100 in 1× PBS at RT for 10 min, blocked overnight with 20% BSA in 1× PBS at 4 °C, and labeled with 1:25 primary antibody α-p24 (Sino, Cat No. 40243-RP01) for 1 h at RT, followed by secondary antibody labeling with 1:250 α-rabbit Alexa Fluor 647 (abcam, Cat No. ab150079) for 1 h at RT. Slides were DAPI stained using 50 µg/mL DAPI for 2 min and treated with 1× True Black Blocker (Biotium, Cat No. 23007) for 30 s. Slides were cover slipped with Vectashield Mounting Media (Vector, Cat No. H-1000) prior to imaging. Control samples were stained with the same conditions described above, and for additional controls, HIV-1 negative cells/tissues were stained with DAPI and secondary antibody only (not shown).

### Image acquisition

Confocal images of cell-associated fluorescence were acquired using the Zeiss LSM 800 confocal system (Carl Zeiss Microscopy, Germany) via the Airyscan super resolution mode. Three laser lines, 405 nm (blue, for nuclei), 488 nm (green, for leukocyte surface antigens [CD45 or CD4 in liver, LN, and spleen; CD68 in brain] and 647 nm (red, for HIV-1 protein, p24) were used. Blue, green, and red signals were separated by a quad DAPI/FITC/TRITC/Cy5 dichroic beam splitter and further acquired using a Gasp detector. A Plan-Apochromat 63x/1.4 Oil DIC objective was used to visualize multi-color labelled cell/tissue samples. ZEN Blue 2.3 software (Carl Zeiss Microscopy, Germany) was used to generate original images. All images were acquired under the same instrument settings. Signal to noise ratio was accounted for by averaging data all images acquired using the same gain offset, detector, and laser excitation power. Saturated signal was avoided by using the software-controlled range for a minimum pixel saturation.

### Peripheral blood viral load

Mice were periodically bled retro-orbitally. RNA was extracted from plasma using a QIAamp Viral RNA Mini Kit (QIAGEN, Cat. 52904) as per the manufacturer's instructions. HIV-1 RNA was converted to cDNA using a SuperScript III First-Strand Synthesis SuperMix Kit (Invitrogen 18080-400) as per the manufacturer's instructions. HIV-1 cDNA was amplified and quantified using qPCR with the following protocol: a single cycle at 50 °C for 2 min, a single cycle at 95 °C for 15 min, 40 cycles at 94 °C for 15 s, 58 °C for 30 s, and 72 °C for 30 s, followed by a single cycle at 72 °C for 30 s using a BioRad LightCycler and BioRad iQ5 software. HIV-1 RNA data were graphed using GraphPad Prism 9 for each mouse (n = 3 per treatment; randomly chosen), along with an average for each treatment.

### DNA standard curve

To generate a standard to quantify HIV-1 DNA copies via qPCR, HIV-1 *gag* amplicons were cloned using a TOPO™ TA Cloning™ Kit (Invitrogen, Cat. 450640), transfected into One Shot® TOP10 *E. coli* (Invitrogen, Cat. C404004), grown on an LB agar plate containing 50 µg/mL kanamycin, and DNA extracted from overnight cultures using a QIAprep® Spin Miniprep Kit (QIAGEN, Cat. 27104). Plasmid DNA was sequenced to determine HIV-1 *gag* integrity and serially diluted for standards [38].

Standards were qPCR amplified alongside mouse tissue DNA samples using primers specific to either HIV-1 *gag* (at 30,000, 3000, 300, 30, and 3 standard copies) or human β-globin (at 100,000, 10,000, 1000, 100, and 10 standard copies). Cycle thresholds were plotted against initial template quantity on a log scale to create standard curves [38]. DNA sample thresholds were plotted against the standard curves to quantify DNA copy number.

### HIV-1 gag DNA quantification in tissue.

Viral DNA loads in mouse tissue were quantified by a qPCR assay measuring total viral DNA (integrated and unintegrated, linear, and circular forms). DNA was extracted from mouse tissue (brain, liver, spleen, and lymph node) using a QIAGEN DNeasy® Blood and Tissue Kit (QIAGEN, Cat. 69506) and analyzed using the QuantStudio 3 PCR system (Applied Biosystems) with 10 µL of genomic DNA in each sample. Samples were analyzed along with the set of standards for both HIV-1 *gag* DNA and human β-globin DNA in 96-well plates in triplicate. DNA copy numbers were quantified with the QuantStudio 3 PCR system using the standard TaqMan protocol. This consisted of a single cycle at 50 °C for 5 min, a single cycle at 95 °C for 10 min, and 45 cycles at 95 °C for 15 s and 60 °C for one minute. All qPCR experiments were repeated three times for reproducibility. Unknown HIV-1 *gag* or human β-globin DNA copy numbers were determined by extrapolation from the known standard DNA copy numbers for HIV-1 *gag* DNA or human β-globin. We expressed HIV-1 *gag* copies as copies per 10^6^ cells. All statistics were calculated using GraphPad Prism 9.

## Results

Hu-mouse tissue samples were selected based upon our previous study [23] where we demonstrated successful CD34+ cell engraftment in NSG mice, verified by flow cytometry identification of human CD45+, CD4+, and CD8+ T cells. Previous data showed an average of 64% engraftment of CD3+ T cells, 38% engraftment of CD4+ T cells, 10% engraftment of CCR5+ T cells, and 23% engraftment of CD8+ T cells, on average, within the total population of CD45+ cells [23, 32]. Flow cytometry data for human cells present in hu-mice were normalized with respect to CD45+ cell numbers. Viral load measurements from infected mice confirmed stable, productive HIV-1 infection reaching a maximum of 5 × 10^5^ to 5 × 10^6^ copies/mL at around week 3 post-infection. This load was generally maintained through week 20 post-infection [14, 18, 23].

**Fig. 1:**
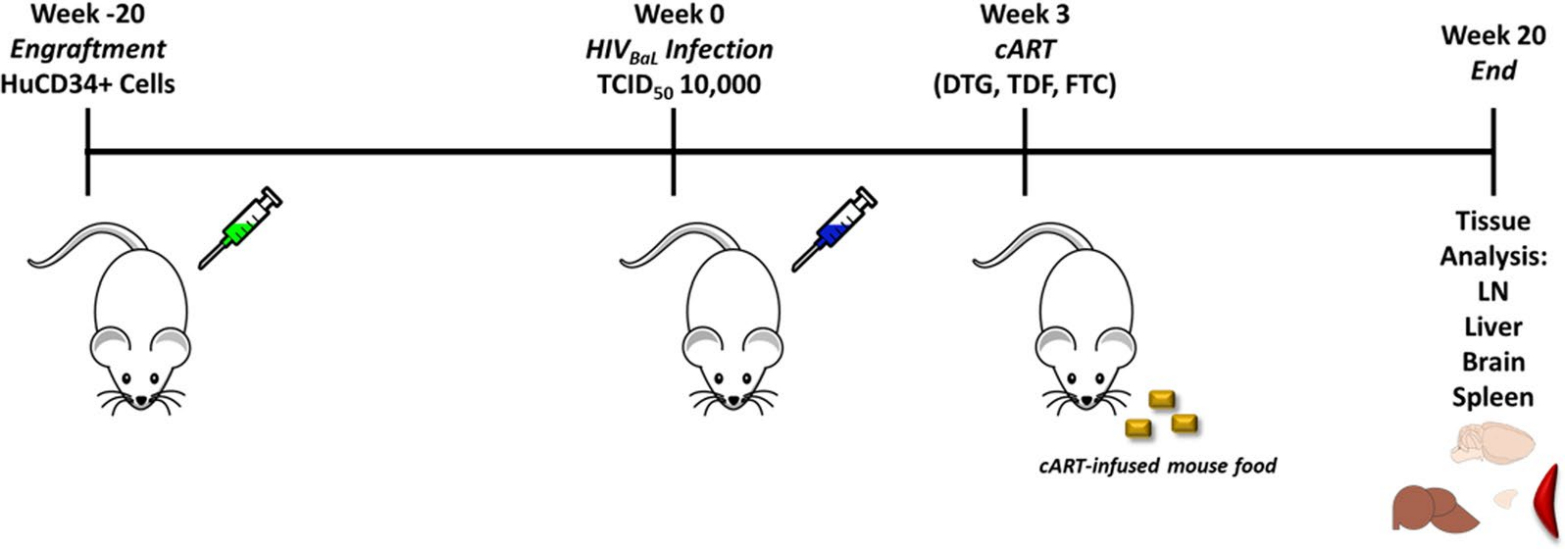
Timeline of experimental steps. Newborn mice were irradiated and injected with human CD34+ hematopoietic stem cells. After 20 weeks, engrafted mice were infected with 10,000 TCID_50%_ HIV-1 BaL and a subset treated with cART 3 weeks post-infection. This subset of HIV-1 infected mice received oral cART containing Dolutegravir (DTG), Tenofovir Disoproxil (TDF), and Emtricitabine (FTC) for 17 weeks. After 20 weeks post-infection, mice were euthanized, and organs collected for qPCR and imaging experiments. Total DNA was quantified using qPCR in all four collected tissues, and infection sites mapped via p24 imaging in all four collected tissues using super resolution Airyscan imaging

The timeline is represented in Fig. 1. At 20 weeks prior to viral challenge, 13 new-born NSG mice were irradiated (100 cGy), then engrafted with 1 × 10^5^ human CD34+ hematopoietic stem cells each and evaluated 20 weeks later for engraftment by flow cytometry, with the criteria that CD3+ cell numbers must be greater than CD19+ cell numbers. Eight mice were infected intraperitoneally with 10,000 TCID_50_ HIV-1 BaL [34] at week 0 (Fig. 1) with 5 mice remaining as uninfected controls. Random mice were bled and evaluated for viral RNA, as well as human cell numbers to confirm productive HIV-1 infection (Fig. 3). At week 3, infected mice were split into an untreated group (3 mice) and a cART-treated group (5 mice). cART therapy was initiated and orally administered once daily, in the form of food pellets, until the endpoint at week 20 where they were evaluated for viral RNA (3 randomly chosen mice per treatment group) and intracellular DNA.

### Visualization of HIV-1 p24 protein in HIV-1 infected hu-mice to demonstrate productive HIV-1 infection

**Fig. 2:**
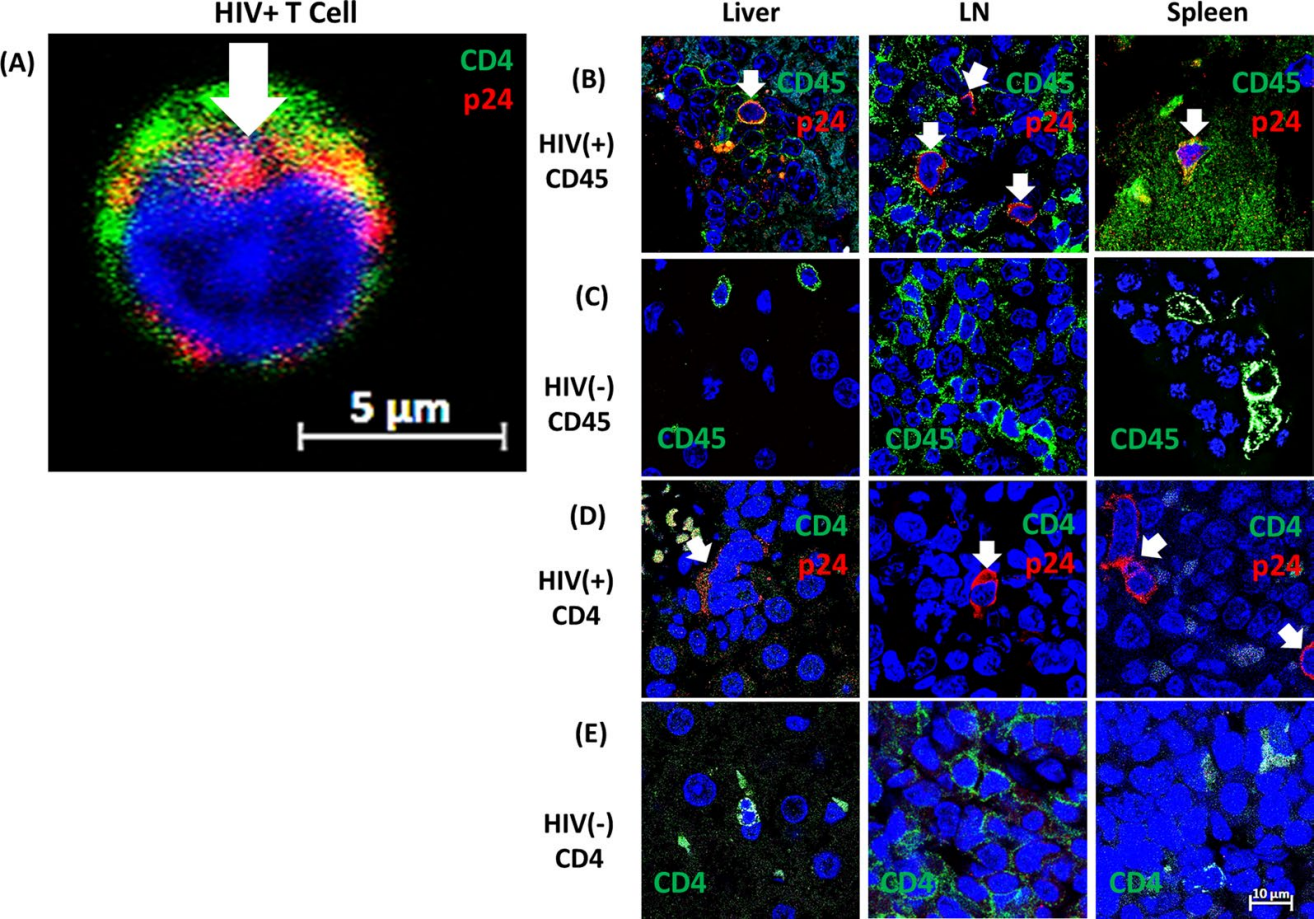
Confocal images of p24 in CD45+ lymphocytes or CD4+ T cells from hu-mouse tissues. Shown are a donor PBMC infected in vitro (**A**) and HIV(+) CD45+ (**B**), HIV(−) CD45+ (**C**), HIV(+) CD4+ (**D**), or HIV(−) CD4+ (**E**) lymphocytes or T cells in mouse liver, lymph node, and spleen from infected or uninfected hu-mice. Representative tissue sections were analysed by α-p24 mAb in red and human α-CD45 or -CD4 in green among the indicated groups of mice. White arrows indicate cells expressing p24 (**A**, **B**, and **D**). The bar size is 5 µm for PBMCs in panel **A** and 10 µm for tissue sample cells in panels **B**–**E**

To confirm CD34+ cell engraftment and productive HIV-1 infection, we analyzed HIV positive and HIV negative mouse livers, spleens, and LN tissues for the presence of human T cells and for expression of viral p24 by Airyscan confocal microscopy before proceeding with functional assays. In Fig. 2A, a single T cell infected in vitro represents the positive control for cytoplasmic p24 (red fluorescence) as well as α-huCD4 Ab (green fluorescence). The nucleus is blue from the nuclear dye DAPI. In vitro optimization of the staining procedure was applied to the following tissue staining experiments with 754.34 PE mol/cell; PE being the fluorophore for FACS evaluation of surface CCR5. As shown in Fig. 2B, D, cell-associated p24 was detectable, as indicated by white arrows, in LN, spleen, and liver of HIV-1 infected hu-mice.

We previously showed successful engraftment with huCD45+ and CD4+ T cells in peripheral blood samples via flow cytometry [23]. The best-defined HIV-1 reservoirs which persisted during the cART regimens were located inside the latently infected CD4+ T cells [39, 40]. We initially focused on visualizing infected CD4+ T cells. However, infected huCD4+ cells were rare in solid tissues from infected mice, suggesting down-regulation of CD4 expression because of HIV infection. In contrast, LN, liver, and spleen of non-infected mice (Fig. 2E, green fluorescence) did contain huCD4+ cells, suggesting either that infection depletes these cells from solid tissue, or that CD4 is downregulated by Nef. However, CD45+ lymphocytes were present that also expressed p24, as clearly shown in Fig. 2B, demonstrating productive HIV-1 infection of lymphocytes. Figure 2B–E present 12 individual images [6 for HIV(+) and 6 for uninfected mice] from three different organs, stitched together via ZEN Blue 2.3 software. Similar results were obtained with intestinal and brain tissues from HIV-1(+) hu-mice (not shown). There was a major difference between the number of cells in Fig. 2D, E because human CD4+ T cells, which are depleted by HIV-1 infection, are the majority (> 60%) of human CD45+ cells in uninfected humanized mice (as previously shown by flow cytometry [23]). We also stained for infected monocytes/macrophages and microglia in brain tissue using CD68 as a marker. Both CD68+p24+ and CD68+p24− cells were evident in infected mice (Additional file 1: Figure S1).

### Reduction of viral RNA in peripheral blood by modified cART

**Fig. 3:**
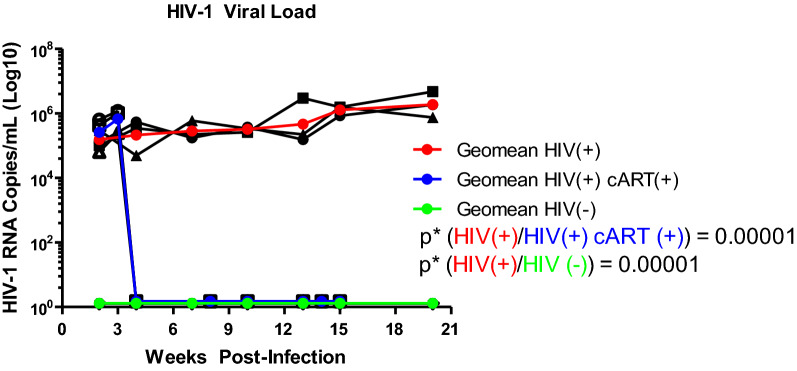
Peripheral blood viral loads. Mice were bled every few weeks and RNA isolated from plasma. RNA copies/mL were calculated from qRT-PCR as described in “Materials and methods”. Copies/mL are graphed on a log10 scale based on weeks post-infection. cART treatment was initiated at 3 weeks p.i. Each treatment group contained 3 mice. A color-coded relative geometric mean for each treatment group was added with HIV(+) in red, HIV(+) cART(+) in blue, and HIV(−) in green

We quantified viral RNA copy numbers by qRT-PCR to demonstrate HIV-1 infection and show reduction in HIV-1 RNA copy numbers by this cART combination (Fig. 3). In HIV-1 infected samples, HIV-1 copies remained relatively consistent at approximately 10^5^–10^6^ copies/mL for up to 20 weeks post-infection. In infected samples treated with the modified cART, HIV-1 copies remained relatively consistent at approximately 10^5^–10^6^ copies/mL until administration of cART at week 3, at which point they rapidly dropped to undetectable and remained there for up to 20 weeks post-infection when cART treatment ended. HIV-1 negative mice had no detectable HIV-1 RNA for the entire 20 weeks, as expected. Each set of data were obtained from randomly chosen animals. The samples were assayed twice to ensure reproducibility, as shown by the black lines. The p values for the ratios are shown in Fig. 3. HIV(+) cART(+) significance compared to HIV(−) could not be calculated due to all end point values equalling zero, but the data sets are identical in their lack of HIV-1 detectability.

### Reduction of viral DNA in tissue by modified cART

We quantified viral DNA copy numbers to assess whether the new cART combination effectively decreased the viral load in solid tissues. We used a qPCR assay originally validated in primary cells [38] to quantify viral DNA in hu-mouse HIV-1 and cART treated tissues. We first compared Ct values of our standard derived from infected hu-mouse tissue with those from ACH.2 cells, which have a single copy of proviral DNA. Agreement of the two sets of values at different input levels showed the method to be accurate (Additional file 1: Figure S2).

**Fig. 4:**
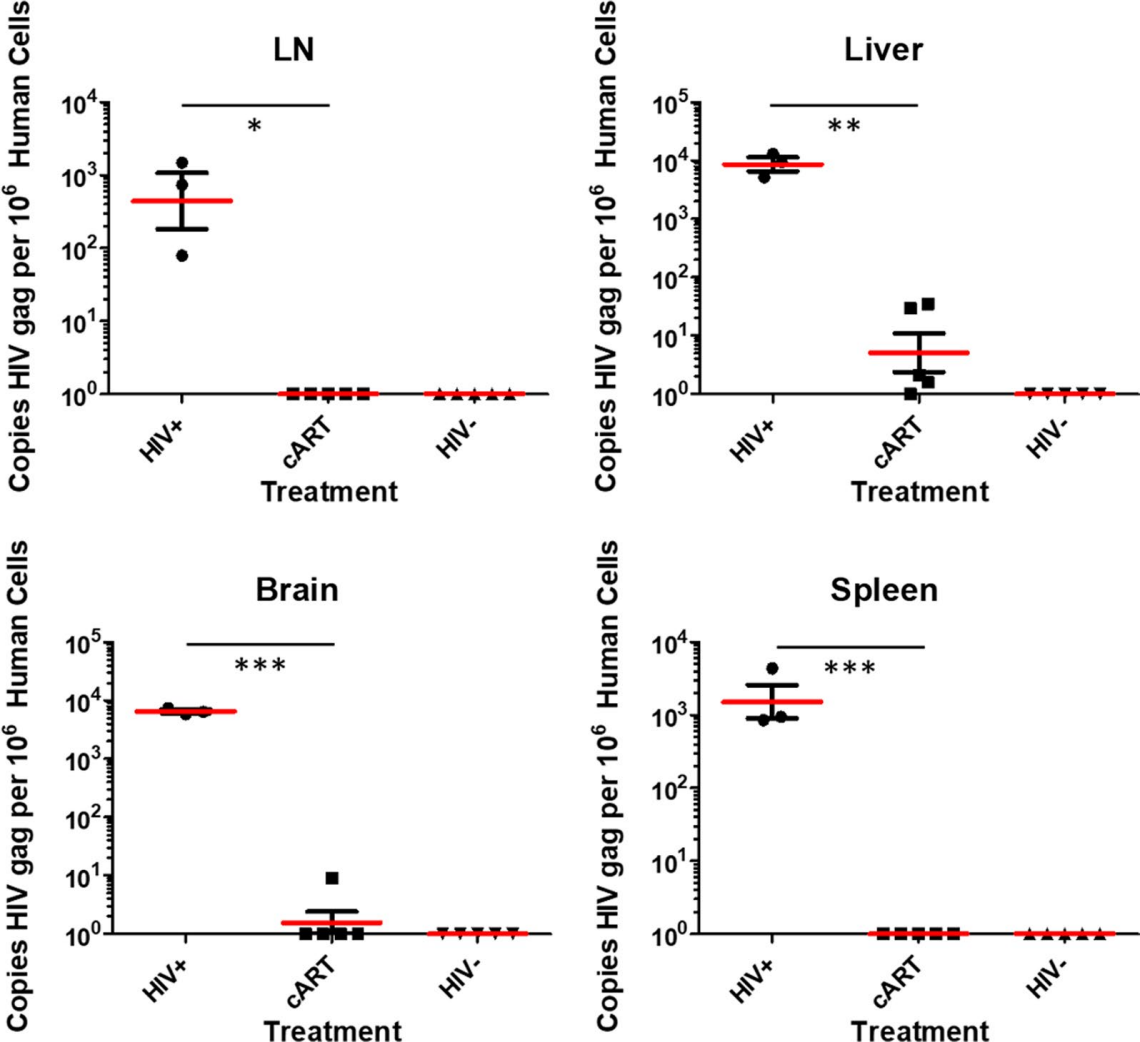
Viral DNA levels in tissues corresponding to visualized productive HIV-1 infection sites in Fig. 2. The first column in each panel represents tissue samples from infected mice; the second column represents tissues from HIV-1 infected, but cART treated mice; and the third column represents tissues from uninfected mice. The first panel is from LN, the second panel from liver, the third panel from brain, and the fourth panel from spleen tissues. The data include groups of 3 animals for the HIV-1 positive group, and 5 different animals for the HIV-1 positive, cART treated group and the HIV-1 negative control group. P values are 0.041, 0.001, < 0.001, and < 0.001 respectively, with the red lines indicating the average value of 3 or 5 mice per group. Copy numbers are graphed on a log_10_ scale and reported as copies per 10^6^ human cells. *p < 0.05, **p < 0.01, ***p < 0.001

Next, we quantified total viral DNA (integrated and unintegrated) in LN, liver, brain, and spleen tissue from infected hu-mice to determine the effect of the cART treatment. The standard DNA curve used in this experiment is described in detail in Additional file 1: Figure S2. The mice were treated orally with the new cART formulation for 17 weeks total after the initial 3-week HIV-1 infection. We also collected LN, liver, brain, and spleen tissues from non-infected controls. Figure 4 shows DNA copy numbers in tissues from these three experimental groups. The first columns of data show viral DNA copy numbers in HIV-1 infected and non-treated mice, the second columns show the cART-treated mice, and the third column the uninfected mice. The infected group contained 3 experimental animals, with a male/female ratio of 2 males:1 female, infected/cART group contained 5 experimental animals, with a male/female ratio of 3 males:2 females. The uninfected group contained 5 experimental animals (3 males:2 females).

The data in Fig. 4 show a striking reduction of viral DNA levels in the solid tissues of mice treated with the new cART combination. The cART was most effective in the LN and spleen tissues of the hu-mice but was also quite effective in the brain and liver, although viral DNA was still detectable in those tissues. The data in Fig. 4 are from three independent experiments using the same mouse tissues per experimental group. Total viral DNA copy numbers in infected but untreated mice were highest for liver (an average of 9275.3 copies per 10^6^ cells). Brain, LN, and spleen contained 6547.1, 767.4, and 902.55 copies/10^6^ human cells respectively. In contrast to infected but untreated tissue, treatment with the new cART showed lower viral DNA copy numbers (11.4 per 10^6^ cells in liver and 1.5 per 10^6^ cells in brain), while no viral DNA was detected in LN or spleen. Residual viral DNA in cART treated liver and brain thus supports the need for further intensification of cART. This could involve including CCR5 targeting drugs, the efficacy of which was shown by our previous results in vitro and in vivo [23, 38].

## Discussion

This study showed that the cART treatment used in our experiments successfully decreased HIV-1 RNA in peripheral blood and DNA numbers in HIV-1 infected hu-mouse tissues such as spleen, lymph nodes, liver, and brain. It further confirms that the hu-NSG mouse model is appropriate for preclinical testing of advanced antiretroviral therapies for their ability to reduce HIV-1 viral replication, particularly since such models provide various accessible tissues at different stages of treatment. We offer additional support for this model by showing visualized, selected sites of HIV-1 infection in different hu-mouse tissues using super resolution Airyscan confocal microscopy and a striking reduction of viral DNA copies in HIV-1 hu-mice treated with a combination of Tenofovir Disoproxil (TDF), Emtricitabine (FTC), and Dolutegravir (DTG) using real time qPCR. We selected this drug combination based on recent evidence of its superior antiviral efficacy in clinical studies [27, 28, 41, 42] compared with previous cARTs. This study could provide the basis for future studies in vivo using treatments such as nano-formulated therapies, which could include enhancement with CCR5 targeting drugs as a possibility to intensify this cART or cytostatic drugs such as tyrosine kinase inhibitors (TKIs) as adjuvants to standard cART options.

There have been several approaches to achieve sustained viral remission, including passive immunization, early initiation of cART, therapeutic vaccination, and immune modulation with clearance of HIV-1 infected cells [43]. Current cART offers sustained and durable HIV-1 suppression [44] that is largely effective in stopping viral replication. However, to date no drug combinations have been able to fully eradicate viral reservoirs in HIV-1 patients. The ability of HIV-1 to establish latent infection causes the formation of these reservoirs in various tissues, including the central nervous system, LN, gut, and bone marrow. Two major factors favoring the existence of viral reservoirs are the longevity of latently infected cells and ineffective drug penetration into some organs (particularly the central nervous system, [45]). Detection and visualization of cryptic sites of HIV-1 infection in tissues are clearly crucial steps towards HIV-1 eradication [46] and will require appropriate animal models with pseudo-human immune systems.

The existing problem in studies with humans and non-human primates are the limitations on sample availability at multiple stages of infection and from differently administered combined antiretroviral treatments. That is easier to manage when working with humanized mouse models. A convenient small animal model for these types of studies is clearly necessary, although we realize that such a model will likely present limitations due to low human cell recovery and thus the number of cells available for thorough and comprehensive analyses. Sample size is somewhat further limited due to mouse body size (20–22 g), but these issues can be addressed by advanced imaging and more sensitive qPCR techniques [47]. However, the main advantage in working with hu-mice is the accessibility to various organ tissues under different treatment options and different therapy timing. Interestingly, Satou et al. demonstrated that there is a dynamic of clonal expansion of HIV-1 infected cells in hu-mice which makes the hu-mouse models more attractive [48]. Lastly, there appears to be less heterogeneity in the humanized mice than in infected individuals, as previous studies have shown thousands of integration sites in humans [48] (although the number of patients analyzed was small). This is relevant to consider, because there are differences in terms of founder virus sequences, routes of initial infections, and duration of HIV-1 infection and treatments that can affect clonal expansion of infected cells and their tissue distribution.

With these considerations in mind, we have used a hu-mouse model for this type of antiretroviral treatment study and determined to what extent the new combination of cART drugs reduces, depletes, or prevents the establishment of viral reservoirs. A great advantage of this human hematopoietic stem cell-engrafted NOD/SCID/IL2R γ null (NSG) model is the shorter time for human cell reconstitution compared with other hu-mouse models [19].

We first visualized HIV-1 infection by showing expression of p24 in selected mouse tissues (Fig. 2 and Additional file 1: Figure S1). We next measured plasma viral loads to demonstrate cART efficacy (Fig. 3) and quantified total viral DNA copy numbers in selected solid mouse tissues. Both plasma viral RNA and viral DNA in tissues were greatly reduced by this cART. By super resolution confocal imaging, which allows more precise visualization of p24 within HIV-1 positive cells, we showed multiple sites of productive HIV-1 infection, allowing us to assess HIV-1 suppression by the new cART combination [14, 18]. HIV-1 viral replication, as judged by HIV-1 DNA copies in LN, liver, spleen, and brain, were substantially reduced or eliminated by treatment with this combination for 17 weeks.

Significant efforts have been made to eradicate HIV-1 reservoirs present as latently infected CD4+ T cell subsets within solid tissues [14]. In this study, as judged by total intracellular DNA copy numbers in HIV-1 infected hu-mice, the new current cART treatment is effective in inhibition of viral replication, which reduces the establishment of new reservoirs. Our current study further demonstrates the utility of this hu-mouse model to analyze in vivo HIV-1 infection kinetics and to identify HIV-1 productive tissue by confocal microscopy and functional assays, although one shortcoming is a lack of the full human immune cell spectrum. Additional hu-mouse studies are needed to address how to inhibit clonal expansion of HIV-1 infected cells due to T cell homeostasis.

Brain and liver tissues appeared to contain higher apparent viral loads than LN, possibly because they contained a higher proportion of infected cells, rather than a higher number of infected cells. LN had a higher copy number, but a lower copy/cell number. LN are highly populated with human cells, and due to a relatively short infection period, the infection may not have been able to spread among such a large population before samples were collected and analyzed. The lack of total HIV-1 DNA suppression in the liver and brain tissues of HIV-1 infected hu-mice treated with this combination of cART drugs (Fig. 4), indicates the need for further cART drug intensification strategies, perhaps by inclusion of CCR5 targeting drugs.

## Supplementary Information

**Additional file 1: Figure S1.** Confocal images of p24 in CD68+ macrophages from hu-mouse brain. Shown are an HIV(+) CD68+ (**A**) and HIV(−) CD68+ (**B**) macrophages in brain tissue from infected or uninfected hu-mice. Representative tissue sections were analysed by α-p24 mAb in red and human α-CD68 mAb in green among the indicated groups of mice. White arrows indicate cells expressing p24 (**A**). The bar size is 10 µm for tissue sample cells in panels **A** and **B**. **Figure S2.** Comparison of ACH.2 and hu-mouse Plasmid DNA Standards. Threshold cycle (Ct) measured during qPCR using either total intracellular DNA from ACH.2 chronically infected cells or a plasmid that contains the HIV-1 DNA fragment that was originally isolated from an infected hu-mouse tissue. The Ct at 3000, 300, 30, and 3 copies are shown in each graph, with red bars demonstrating the average value and black bars representing standard error.

## Data Availability

The datasets during and/or analyzed during the current study available from the corresponding author on reasonable request.
