# Supplementary material for: Combined cART including Tenofovir Disoproxil, Emtricitabine, and Dolutegravir has potent therapeutic effects in HIV-1 infected humanized mice
Source: J Transl Med. 2021 Oct 30;19:453. doi: 10.1186/s12967-021-03120-w (PMC8557591; doi:10.1186/s12967-021-03120-w)
Supplement: Supplementary file 1 — Additional file 1: Figure S1. Confocal images of p24 in CD68+ macrophages from hu-mouse brain. Shown are an HIV(+) CD68+ (A) and HIV(−) CD68+ (B) macrophages in brain tissue from infected or uninfected hu-mice. Representative tissue sections were analysed by α-p24 mAb in red and human α-CD68 mAb in green among the indicated groups of mice. White arrows indicate cells expressing p24 (A). The bar size is 10 µm for tissue sample cells in panels A and B. Figure S2. Comparison of ACH.2 and hu-mouse Plasmid DNA Standards. Threshold cycle (Ct) measured during qPCR using either total intracellular DNA from ACH.2 chronically infected cells or a plasmid that contains the HIV-1 DNA fragment that was originally isolated from an infected hu-mouse tissue. The Ct at 3000, 300, 30, and 3 copies are shown in each graph, with red bars demonstrating the average value and black bars representing standard error. [file 12967_2021_3120_MOESM1_ESM.docx]

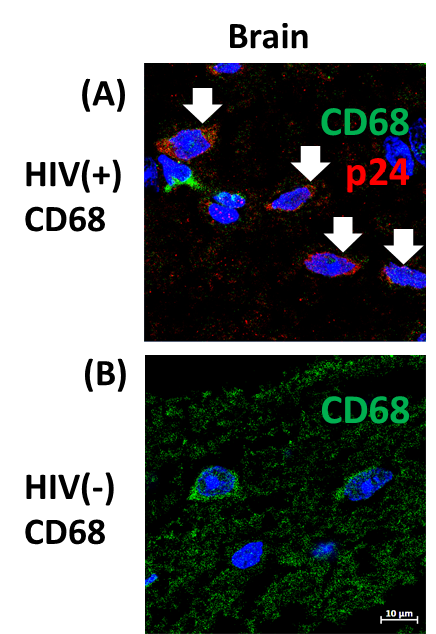
**ADDITIONAL FIGURES**

**Figure S1.** Confocal images of p24 in CD68+ macrophages from hu-mouse brain. Shown are an HIV(+) CD68+ (**A**) and HIV(-) CD68+ (**B**) macrophages in brain from infected or uninfected hu-mice. Representative tissue sections were analysed by anti-p24 mAb in red and human anti-CD68 in green among the indicated groups of mice. White arrows indicate cells expressing p24 (**A**). The bar size is 10 µm for tissue sample cells in panels **A** and **B**.

**
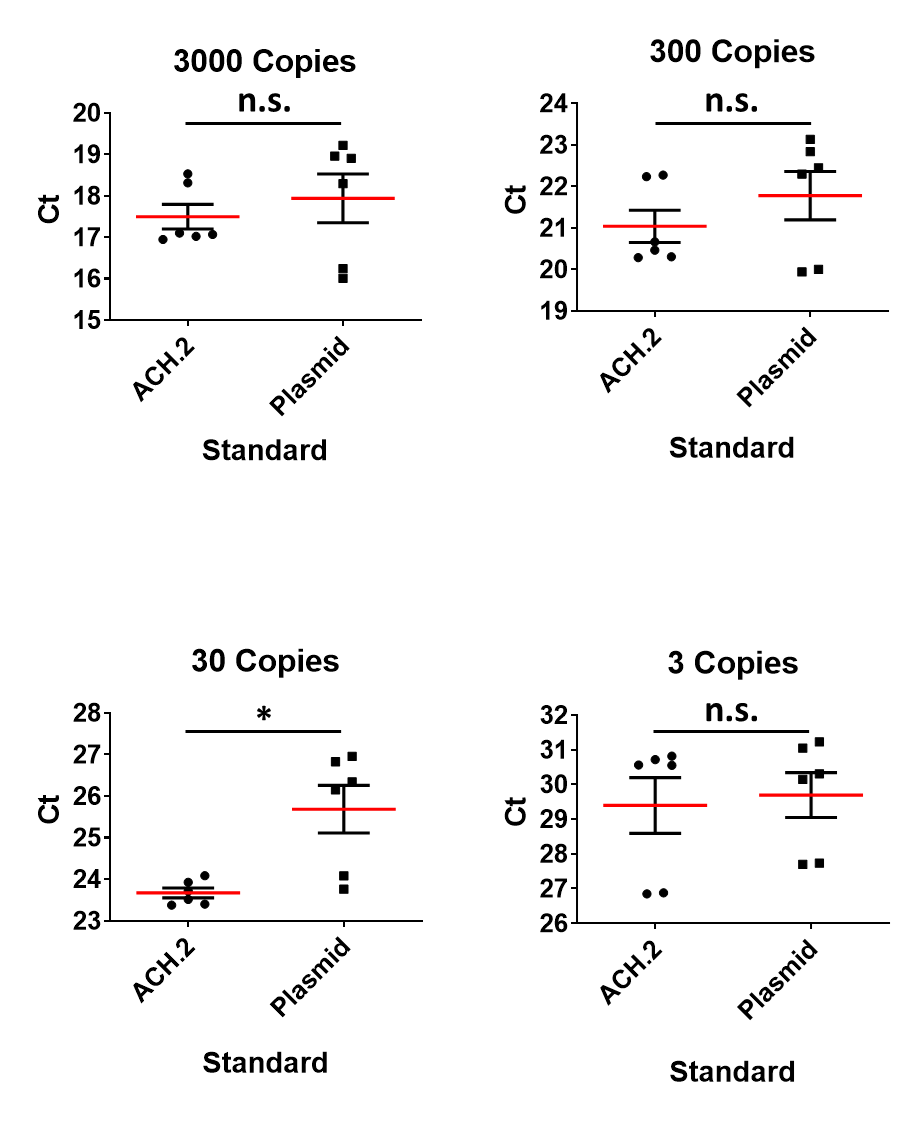
Figure S2.** Comparison of ACH.2 and Hu-mouse Plasmid DNA Standards. Threshold cycle (Ct) measured during qPCR using either total intracellular DNA from ACH.2 chronically infected cells or a plasmid that contains the HIV-1 DNA fragment that was originally isolated from an infected hu mouse tissue. The Ct at 3000, 300, 30, and 3 copies are shown in each graph, with red bars demonstrating the average value and black bars representing standard error.
